# Procedures and Frequencies of Embalming and Heart Extractions in Modern Period in Brittany. Contribution to the Evolution of Ritual Funerary in Europe

**DOI:** 10.1371/journal.pone.0167988

**Published:** 2016-12-28

**Authors:** Rozenn Colleter, Fabrice Dedouit, Sylvie Duchesne, Fatima-Zohra Mokrane, Véronique Gendrot, Patrice Gérard, Henri Dabernat, Éric Crubézy, Norbert Telmon

**Affiliations:** 1 National Institute of Preventive Archaeological Research (INRAP), Cesson-Sévigné, France; 2 University of Toulouse, French National Center for Scientific Research, UMR 5288, Toulouse, France; 3 Unit of Forensic and Anthropological Imaging, Centre Universitaire Romand de Médecine Légale (CURML), Lausanne, Switzerland; 4 Radiology department, CHU Toulouse-Rangueil, Toulouse, France; 5 French Regional Archaeological Service, Rennes, Bretagne, France and French National Center for Scientific Research, UMR 6566, Rennes, France; 6 Medico-Legal department, CHU Toulouse-Rangueil, Toulouse, France; Université de Poitiers, FRANCE

## Abstract

The evolution of funeral practices from the Middle Ages through the Modern era in Europe is generally seen as a process of secularization. The study, through imaging and autopsy, of two mummies, five lead urns containing hearts, and more than six hundred skeletons of nobles and clergymen from a Renaissance convent in Brittany has led us to reject this view. In addition to exceptional embalming, we observed instances in which hearts alone had been extracted, a phenomenon that had never before been described, and brains alone as well, and instances in which each spouse's heart had been placed on the other's coffin. In some identified cases we were able to establish links between the religious attitudes of given individuals and either ancient Medieval practices or more modern ones generated by the Council of Trent. All of these practices, which were a function of social status, were rooted in religion. They offer no evidence of secularization whatsoever.

## Introduction

The evolution of funeral rites in Europe from the Middle Ages to the Modern era is supposedly one of the gradual secularization of certain theological ideas dating from the Middle Ages [1]. Initially, the way in which bodies were dealt with, including embalming, was a religious matter reserved for medieval kings [2]. The practice, it is argued, progressively spread to the nobility. Today, bodies are embalmed for public display, and the social and religious character of the practice is absent. While there is ample historical evidence to support this view [3–7], archaeological data, which could provide a more anthropological perspective, remains wanting [8–10]. Bio-archaeologists have generally seen surgical interventions on the bodies of Renaissance and Modern era European nobles as preparation for the display of the deceased's embalmed remains or as a means to preserve the bodies [11–16]. In developing this interpretation, they have drawn on a contemporary frame of reference, even though religious beliefs a century after the Council of Trent, in particular those associated with the burial of kings, were still fraught with magic and ritual, which was not to disappear until the French Revolution [7]. These interpretations have now been thrown into question by an exhaustive dig carried out in a Renaissance convent in Brittany and the discovery of perfectly preserved bodies and hearts that have been autopsied and subjected to imaging. In their stead, we propose a new view of the history of death in Europe, and a new interpretation of European funeral rites.

## Materials and Methods

### Excavation

In the 17^th^ century, the city of Rennes was the seat of Brittany's parliament and home to a large number of nobles who were attached to the French king. With a population estimated at 45,000, it was the largest city in Brittany, the province that accounted for 10% of the population of the kingdom [17]. The Jacobin convent was the principal burial site for the parliamentary aristocracy [18]. Founded in 1368, it is one of the later mendicant convent in Brittany [19]. It was situated to the west of what is today the Place Sainte-Anne (48°6’54.532" N, 1°40’53.548" O), outside the Medieval and Modern age walls, on the outskirts of the city of Rennes.

There were two burial periods: one spanning the 14^th^ and 15^th^ centuries, the other the 16^th^ through 18^th^. We studied 133 subjects from the first period located at the original chapter house, the garden in the cloisters, and areas around buildings. We did not find any children under four, which does not reflect the natural mortality. The disproportionate number of men was largely due to a mass grave that held more than thirty males aged 30 to 50, in all likelihood soldiers, given the bone injuries that had not yet healed. Because of their location within the convent, other buried remains were deemed to be those of clergymen. The remaining bodies were almost certainly parishioners. Because there is no indication that any of the skeletons from this period was embalmed (0/133), they have not been included in this study.

**Fig 1:**
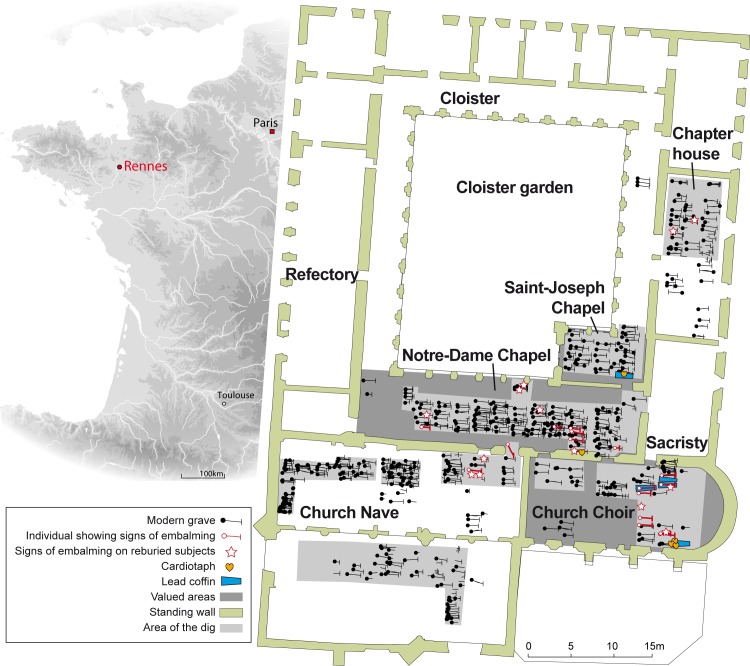
Location of the French city of Rennes and of the 16^th^ through 18^th^ century graves at the Jacobin convent in Rennes.

**Fig 2:**
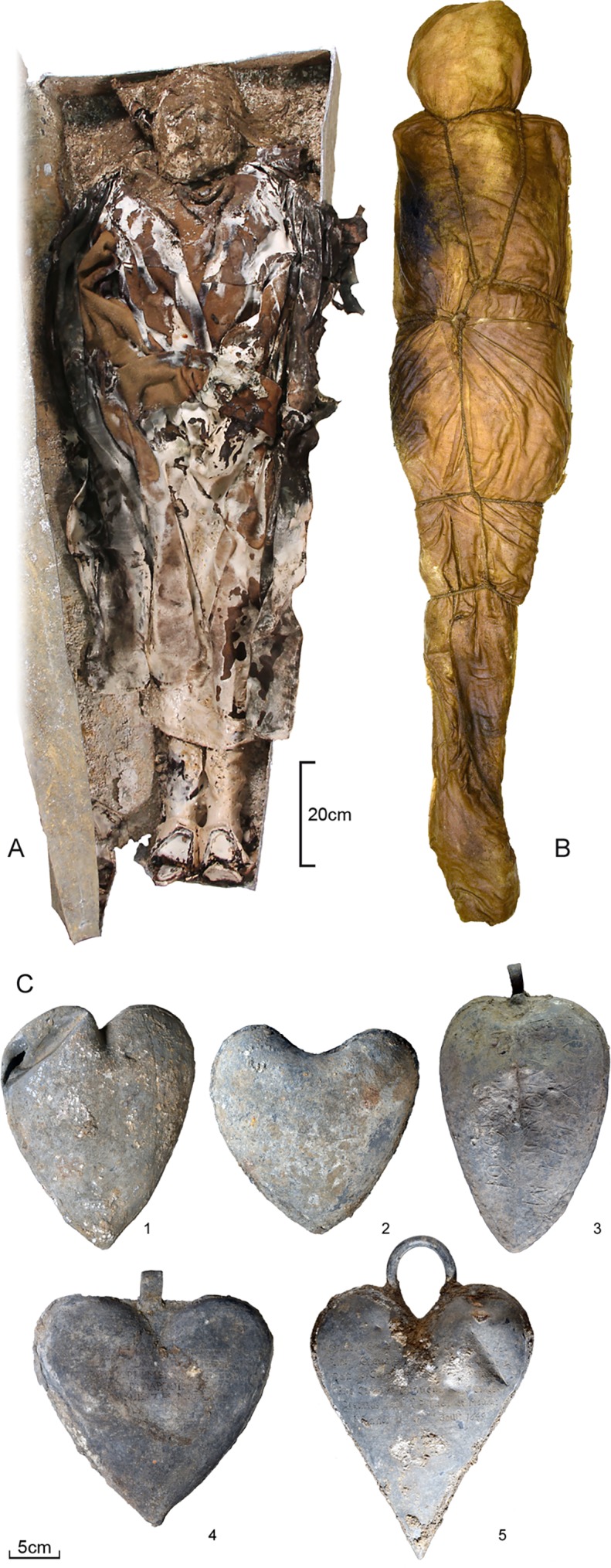
Materials. (A) The body of Louise de Quengo in her lead coffin. (B) The body of Louis Bruslon du Plessis wrapped in two cloth shrouds tightly held in place with a rope. (C) Lead urns containing hearts. Inscriptions: (1) None; (2) "*[…] erine […] tournemine […] / […] D […] vaucler […] / juillet 1584*"; (3) "♥ *DU FIS DE MR DE LA BOESSIE/RE /1626/* "; (4) "*[…] ESTRE […] ANT DE LA PORTE / […]ONSME DV […] E […] SIDANT / […] PARLEMENT […] BRETAIGNE / […] R DARTOI […] / […] LE 7me MAY 1655*"; (5) "*Cy git le Coeur de [*…*] Toussainct de / Perrien Chevalier de Brefeillac &c / Dont le Corps repose [*…*] Sauveur / Pres Carhay Convent des Carmes / Deschaus qu'il a fonde et mourut / à Rennes le 30me aouft 1649*".

The second period involved approximately 1,250 interred subjects, 483 of whom were examined in depth. In addition to the bodies buried in wooden coffins or the ground, we unearthed five coffins and five urns containing hearts, all ten of them made of lead (Fig 1). The urns were heart-shaped, and three of them had suspension eyes (Fig 2C). Three of the five urns were found in the church choir, at the head of a lead coffin, one was resting on a lead coffin in a side chapel, the third lay by itself in a pit near a recess tomb in a side chapel. With the exception of one underrepresented group, children aged four or younger, the composition of the buried subjects from this second period (406 adults, 56,5% of them male, 43,5% female, and 77 children) corresponds to the natural mortality rate. We were able to compare archaeological data from this period with archival references to the burials of 113 subjects, 74% of whom were nobles (84/113), 8% clergymen (9/113), and 4% (4/113) of the Third Estate. The remaining 14% (16/113) were of uncertain social status but most likely of the Third Estate as well. On the basis of archival entries and the inscriptions on the lead coffins and heart-shaped urns, we were able to identify three of the bodily remains and four of the hearts, all of which were those of persons of nobility. We also studied the body of Louis Bruslon du Plessis, who died in 1661 and thus falls within this second period. His was the only body that was unearthed elsewhere, under the entrance to the Jesuit Toussaints Church in Rennes (48°6’32.872" N, 1°40’32.508" O) that is located about half a mile from the Jacobin convent. He was included in this study because of both the excellent state of preservation of the body and the post-mortem interventions it had undergone (Fig 2B), which allowed for comparisons with those of the Jacobins.

### Disinterment, Treatment and Anthropological Analysis

The graves were the object of an archaeological dig and a study carried out by anthropologists [20]. Sex could be assumed through visual examination of the os coxae [21] and through measurements of the pelvis (referred to in French as "probabilistic sex diagnosis" [22]). When the os coxae were not satisfactorily preserved, measurements of the long bones enabled us to provide a secondary sexual diagnosis. For the adults, age at the time of death was estimated through observation of the sacropelvic surface [23] and for children we studied the stages of tooth mineralization [24,25], as well as bone maturation when dental data were insufficient [26–29]. The examination of the urns and the intact bodies was undertaken by a multidisciplinary team and limited to 72 hours, so as to avoid the loss of information that a reactivated process of decomposition might have triggered. Bodies that were clothed were subjected to computed tomodensitometric evaluation for the recording of data and lesions [30] before being undressed, layer by layer. To avoid contamination, the heart-shaped urns were immediately placed in hermetically sealed containers and stored in freezers at a temperature of -18°C, to await examination. There was one exception: an urn that seemed perfectly preserved was maintained in a refrigerated environment at 6 C°. Once removed from their containers, the organs were studied under usual forensic medicine methods (autopsy, macroscopic examination, histology) and high resolution medical imagery such as tomodensitometry and magnetic resonance prior to and after coronary opacification [31].

### Radiology

Multislice computed tomography (MSCT) was performed in Toulouse in the Radiology Department (Sensation, Siemens, Erlangen, Germany). Slice thickness was 16*0.75 mm, with 0.75 mm collimation and a 512*512 matrix. Post-processing was performed on a Leonardo console (Siemens, Erlangen, Germany) with Osirix software. Reconstructions included two-dimensional multiplanar reconstruction (MPR) and three-dimensional maximum intensity projection (MIP) and volume rendering technique (VRT).

### MSCT and Autopsy

Full-body, computed tomography was performed on the head, neck, chest, abdomen, pelvis, and upper and lower limbs. The images were interpreted by a board-certified radiologist. The case was treated as a forensic case. So study allowed for an exhaustive examination of lesions and sought to determine, where possible, the manner of death. External examinations, autopsies and anthropological studies were carried out by anthropologists. A complete autopsy was performed. All three cavities (cranial, thoracic, abdominal-pelvic) were examined.

## Results

**Fig 3:**
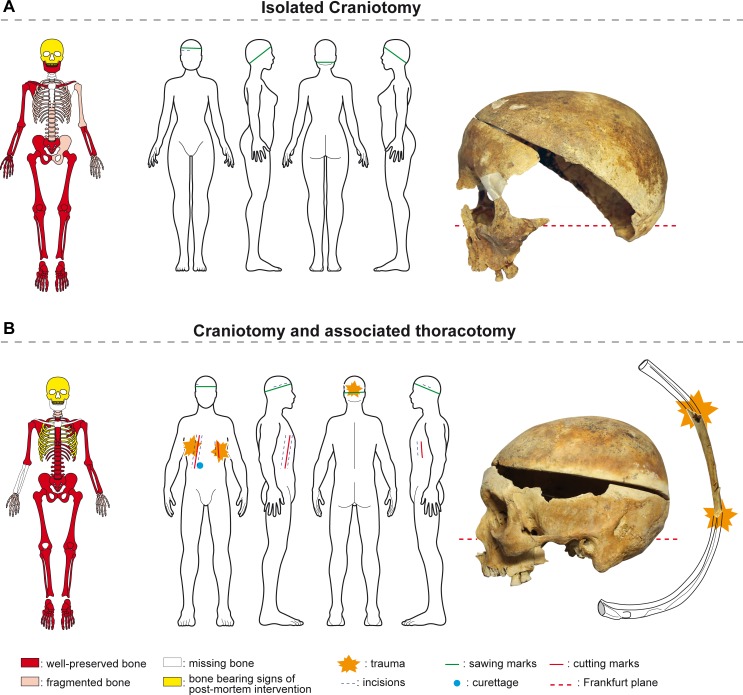
Bones lesions. Representation of skeletal preservation, anatomical location, types of lesions and bone pictures. (A) Isolated suboblique craniotomy (grave 1209). (B) Subhorizontal craniotomy and associated thoracotomy, cut marks on the lower edges of the 5^th^ left rib with secondary fractures (grave 1226). **Sawing** is attested by tooth marks and / or scrapping marks. The deep **cutting grooves** evidence crushing and repetitive attempts at severing the ribs and / or sternum using pruning shears or choppers. The fine grooves that can be observed on the external side of the bones are due to scalpel **incisions** in order to remove teguments. Those on the internal side appear as a consequence of **curettage** the ribs and / or os coxae.

Eighteen objects of study (12 complete skeletons, 1 corpse, and 5 hearts) out of the 483 from the 16^th^-18^th^ centuries and 17 bones out of the 5,940 skeletal remains from secondary deposits (in filling of graves) or from partial skeletons found in graves that were not well-preserved showed signs of post-mortem intervention in the form of craniotomies, and/or an opened thoracic cage or abdomen (Table 1), and/or heart extraction (Fig 3).

**Table 1:** Inventory of post-mortem cuts and / or saw marks on bones

Grave	Age group	Sex	Inhumation	Housing	Site	Skull	Thorax	Pelvis
71	[5–9]	undetermined	Primary (complete)	Wooden Box	Notre-Dame	1	0	0
106	> 50	Male	Primary (complete)	Wooden Box	Notre-Dame	1	0	0
1000	[5–9]	undetermined	Primary (complete)	Wooden Box	Church (Choir)	1	0	0
1189	> 40	Female	Primary (complete)	Wooden Box	Church (Choir)	1	0	0
1209	> 20	Female	Primary (complete)	Wooden Box	Church (Choir)	1	0	0
1008	> 40	Male	Primary (complete)	Lead Coffin	Church (Choir)	1	1	0
1184	[20–49]	Male	Primary (complete)	Wooden Box	Church (Passageway)	1	1	0
1202	[20–49]	Male	Primary (complete)	Wooden Box	Church (Choir)	1	1	0
1226	> 40	Male	Primary (complete)	Wooden Box	Church (Choir)	1	1	0
168	[20–49]	Male	Primary (complete)	Pit	Notre-Dame	0	1	0
212	[20–39]	Male	Primary (complete)	Wooden Box	Notre-Dame	0	1	0
1004	[20–29]	Female	Primary (complete)	Lead Coffin	Church (Choir)	0	1	0
102	[5–14]	undetermined	Primary (partial)	Wooden Box	Notre-Dame	1	-	-
130	> 60	Female	Primary (partial)	Wooden Box	Notre-Dame	-	1	0
1229	> 20	undetermined	Primary (partial)	Wooden Box	Church (Choir)	1	-	0
1252	> 50	Female	Primary (partial)	Wooden Box	Church (Nave)	1	-	0
49	> 20	undetermined	Secondary		Notre-Dame	1	-	-
73	> 30	undetermined	Secondary		Notre-Dame	-	-	1
288	> 20	undetermined	Secondary		Notre-Dame	1	-	-
288	> 20	undetermined	Secondary		Notre-Dame	1	-	-
706	> 20	undetermined	Secondary		Chapter house	1	-	-
1000	> 20	undetermined	Secondary		Church (Choir)	1	-	-
1004	[20–49]	Male	Secondary		Church (Choir)	-	1	-
1248	[5–9]	undetermined	Secondary		Church (Nave)	1	-	-
1252	> 20	undetermined	Secondary		Church (Nave)	1	-	-
1252	> 20	undetermined	Secondary		Church (Nave)	-	1	-
6009	> 20	undetermined	Secondary		Notre-Dame	1	-	-
7008	> 20	undetermined	Secondary		Chapter house	1	-	-
8000	> 20	undetermined	Secondary		Church (Choir)	1	-	-

(1 = present; 0 = absent; - = no data).

**Fig 4:**
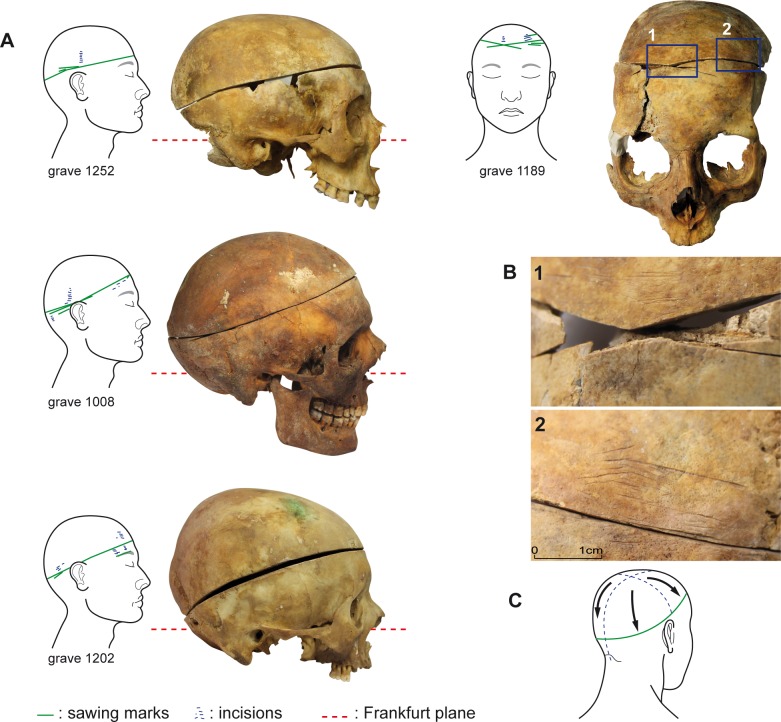
Craniotomies. (A) Examples of subhorizontal to suboblique craniotomies from the Jacobins’ convent. (B) Details of bone lesions from the skull in grave 1189: (1) sawing marks, (2) fine grooves due to the removal of the teguments. (C) Illustration of Dionis’s recommended process (1765).

22 skulls, representing 4.5% (22/483) of the objects from that period, had been opened: 9 from the 249 skulls found in the 483 primary burials and 13 of the 235 complete, isolated skulls. 5 of the 9 well-preserved subjects (1 male, 2 females, 2 children) had undergone only craniotomies, with horizontal to suboblique incisions (**Fig 4**). 4 of these 5 were found in the choir, which was an area of the church highly regarded by the Catholics of the time. In the case of four other craniotomies, all of them performed on males, there were signs that the thorax had been opened as well. These three were also found in highly valued locations: the choir and the passageway leading to Notre-Dame Chapel (Fig 1).

**Fig 5:**
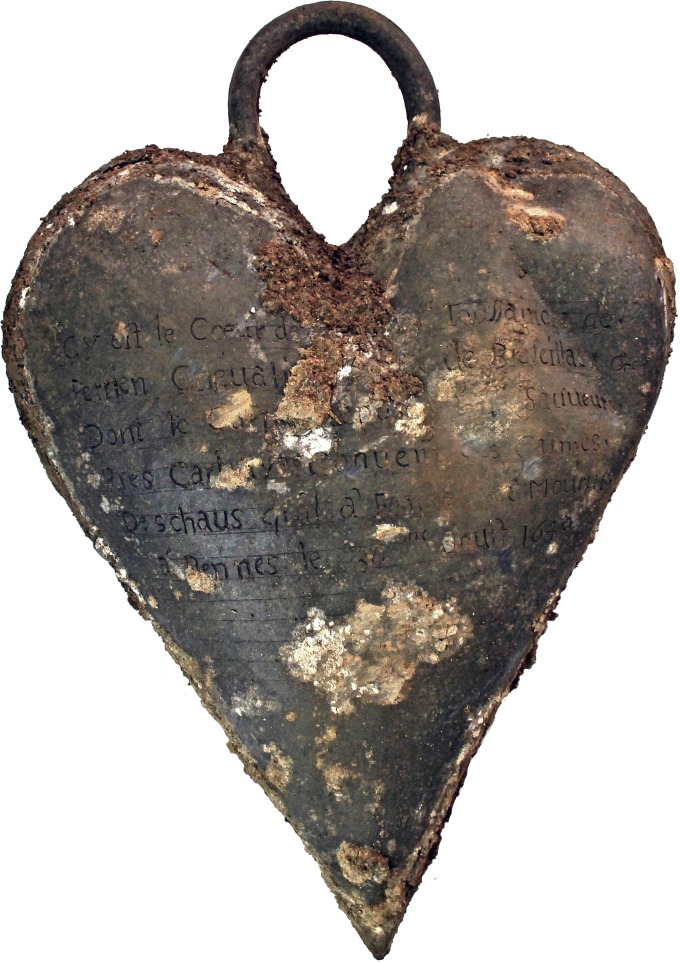
Toussaint de Perrien's cardiotaph. The epitaph gives the deceased's identity, the location of his tomb, and the date of his death: "*Cy git le Coeur de [*…*] Toussainct de / Perrien Chevalier de Brefeillac &c / Dont le Corps repose [*…*] Sauveur / Pres Carhay Convent des Carmes / Deschaus qu'il a fonde et mourut / à Rennes le 30me aouft 1649*" containing hearts. ["Here lies the heart of Toussainct de Perrien, Knight of Brefeillac &c / whose body lies […] Savior / Near Carhaix (in the) Discalced Carmelite Convent / which he founded, and who died in Rennes on August 30, 1649"].

**Fig 6:**
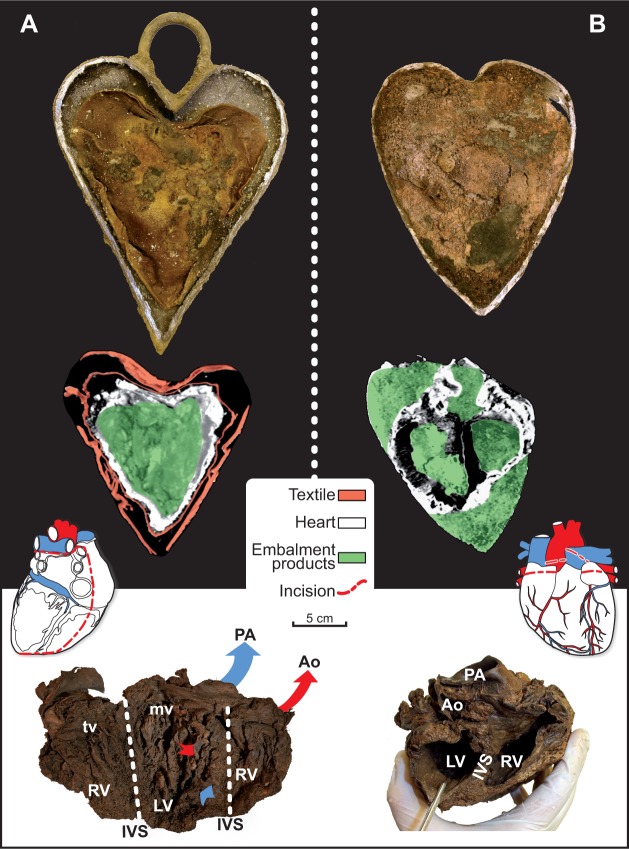
Protocol for Embalming Hearts. The major blood vessels were severed at the point where they exited the heart. The heart was covered with either grain or vegetable fiber that was also inserted into the ventricles. Two of the four well-preserved hearts had been embalmed, with incisions made in each of the atria to allow the ventricles to be padded individually, thereby maintaining as much as possible their original shape (A). The right ventricles of the other two hearts had been slit open from the apex to the top of the atrium, after which the interventricular septum was cut, thus merging the two ventricular cavities, which were then filled with either fiber or grain or both (B). PA: pulmonary artery; Ao: aorta; LV: left ventricle; RV: right ventricle; IVS: interventricular septum; tv: tricuspid valve; mv: mitral valve.

Four of the heart-shaped urns bore inscriptions dating between 1584 and 1655, indicating that the practice of placing hearts in urns had been engaged for some 70 years (Fig 2C2 to 2C5). The three cardiotaphs unearthed at the foot of a lead coffin in the church choir dated from 1584, 1626, and 1685; the two more recent of these had been fitted with suspension eyes. The urn found resting atop a lead coffin was that of Toussaint de Perrien, knight of Brefeillac, who died on August 30, 1649 (Fig 5). The fifth bore neither inscription nor suspension eye. It was found in a pit located at the foot of a recess tomb in a side chapel. Only four of the five hearts contained in urns were well-preserved and showed signs of embalming. After extraction from the thoracic cage, the four had been embalmed with vegetable matter. Two of them were found in hemp sacks, one of which was wrapped in tow (Fig 6).

**Fig 7:**
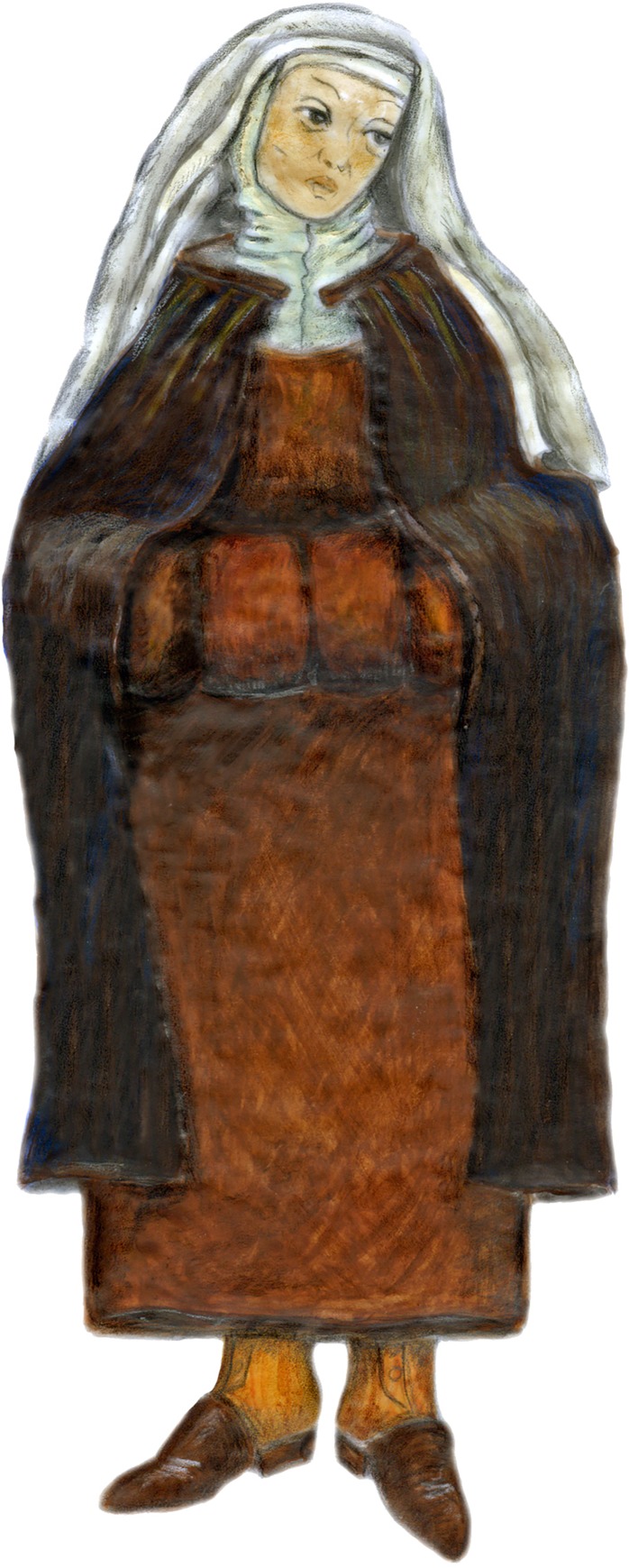
Rendering of Louise de Quengo. She is dressed in religious apparel: a black cloak, a brown monk's cloth dress of coarse twill wool, a large scapular, a shirt of un-dyed twill wool, gaiters, woolen chausses, and leather mules with cork soles. There are several veils on her head held in place by a toque, as was the custom of nuns of that time.

One of the lead coffins was that of a noblewoman, Louise de Quengo, a benefactor of the church who was more than 65 years old when she died on March 10, 1656 (Fig 2A). The very simple religious (Fig 7) attire attests to a desire to be associated with a church dedicated to the poor, as was the case for the Jacobin convent that was run by one of the mendicant orders, the Dominicans. The heart of Toussaint de Perrien, her husband, was set on top of her coffin. He had died seven years before her and had been buried 125 miles from Rennes, in a convent that he had founded. The three cardiotaphs found atop lead coffins in the church choir belonged to Catherine de Tournemine, Monsieur d'Artois, and the son of la Boessière. The burial sites of these three people are unknown to us. As with Louise de Quengo, we do not know if this is a case of spouses (or other family members) being united after death or whether these three urns had been moved here for reasons unrelated to family ties, which is more likely. The latter hypothesis rests on the dates inscribed on the urns, which stretch over a period of 70 years, and the fact that during the French Revolution lead was recovered to make bullets. The urns found in the graves might have been hidden there by Dominican friars living at the convent.

**Fig 8:**
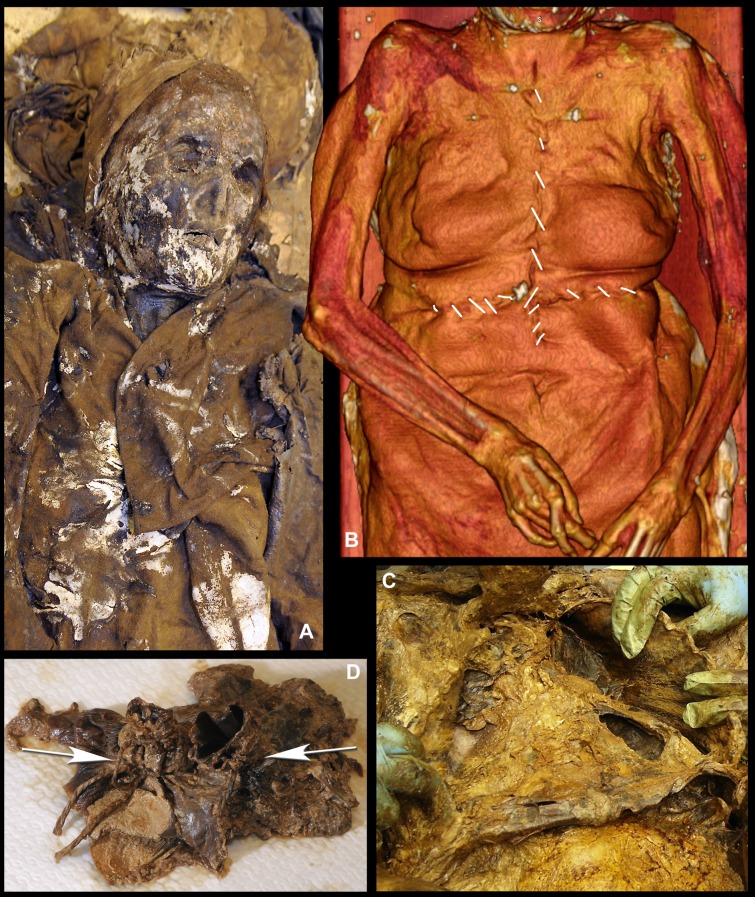
The Post mortem Surgical Operation on Louise de Quengo's Body. (A) View of the top half of Louise de Quengo's body, partially unclothed. (B) The thorax was cut into with two large incisions in the shape of a cross: one extending from the suprasternal notch to just above the navel, the other, perpendicular to the first, beneath the chest, in front of the lower ribs. The integuments were partially folded over to allow access to the sterno-costal cartilage (2 to 12), which was then severed with clean, bilateral cuts. The breastplate was lifted, giving access to the organs and allowing the diaphragm cupola to be severed (C). A vertical cut, 5 cm long, was made on the left side of the pericardial sac (D). Inside the pericardial cavity, the aorta and the pulmonary artery, whose supravalvular portions remained intact, were ligated shut with two separate ties using thread similar to that used to close the incision in the thorax. The principal blood vessels were severed and the pericardial sac emptied of its contents. The breastplate was repositioned, the soft tissues put back in, and the abdominal-thoracic incision crudely sewn closed.

**Fig 9:**
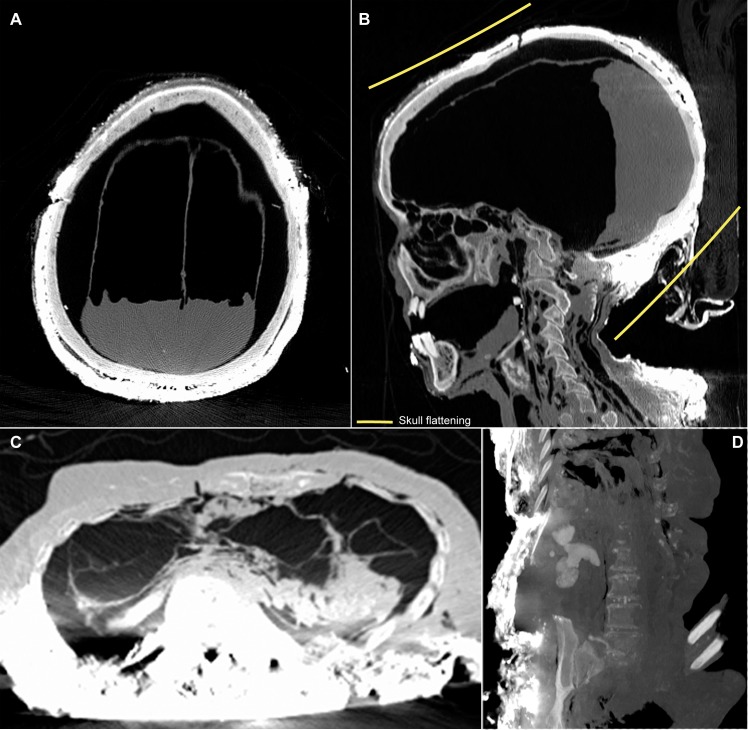
Tomodensitometric examination. (A) The meninges had fallen and begun decomposing but the cerebral parenchyma resting against the posterior section of the cranium is still visible (Skull axial plan). (B) Skull and cervical vertebrae sagittal plan showing evidence of intentional cranial deformation. (C) The pericardial sac is empty. The hyperdense aspect of the posterior thoracic soft tissues as well as the bone structures are visible (axial plan). (D) Dense kidney stones are visible on the abdomino-pelvic coronal plan.

Louise de Quengo's corpse, which weighed 66 pounds, was a natural mummy that had been preserved in the stable, oxygen-free environment of her sealed coffin. As opposed to the other lead coffins, all of which had been pierced, hers came down to us intact. The autopsy revealed that only her heart had been removed (Fig 8) and that the operation had been performed by one or several persons with considerable knowledge of thoracic anatomy. There were no signs of slits on the bones or indications that they had been cut through or into. The computed tomography performed on the body also revealed ante mortem pathologies, as well as funerary manipulations (Fig 9). The MSCT revealed at the cephalic extremity an intentional cranial deformation [32]. The rests of the brain were also visible. The bone structures were completely covered with a thick layer of a hyperdense material. At the cervical stage, some carotid calcifications were visible. The spine was intact. At the chest level, the MSCT revealed that a thoracotomy had been realized, with a bilateral section of the sterno-costal cartilages. Furthermore, the pericardial sac was empty, with the heart absent. A mediastinal and pericardial cut was visible. It was possible to localise all the major thoracic vessels (arteries and veins) which were air filled. The lungs were present, presenting bilateral adherences. At the abdomino-pelvic stage, some arterial calcifications were visible (aorta and internal iliac branches). Some hyperdensities were visible within both kidney parenchyma. The soft tissues of the posterior part of the body and the adjacent bones were hyperdense. However, we noted no traumatic injury that could have been responsible for her death. The incisions on three other subjects (one woman buried in a lead coffin and two men) were limited to the thorax, which would suggest that only the heart had been removed; the procedure generally leaves the inside of the rib cage unscarred. Two of these four subjects from whom hearts were extracted had been laid to rest in valued areas of the convent.

**Fig 10:**
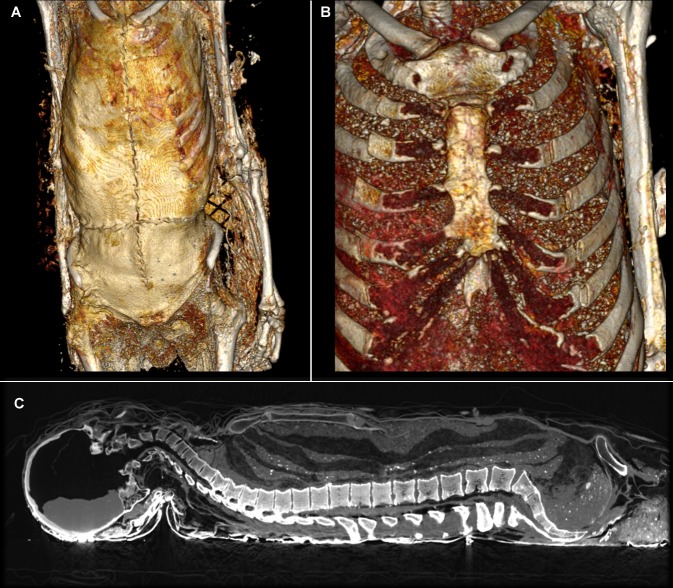
The Embalming of Louis Bruslon du Plessis. (A) The thorax was cut into with two large incisions in the shape of a cross: one extending from the suprasternal notch to just above the navel, the other, perpendicular to the first, beneath the chest, in front of the lower ribs. (B) The teguments were partially folded over to allow access to the ribs (3 to 7), which were then severed with clean, bilateral cuts. The sterno-costal cartilage at the lower end was also severed. (C) The breastplate was lifted, giving access to the thoracic organs, which were completely removed. Of the abdominal organs, only the bladder remained in place. A dozen horizontal layers of tow and vegetable padding were inserted into the whole of the emptied cavities. Once the embalming process was finished, the breastplate was repositioned, the soft tissues put back in, and the abdominal-thoracic incision closed with thirty stitches.

Four subjects, including three males who were buried in the choir of the church, had both their skulls and their thoraxes opened. The complete curettage of the thorax and the abdomen suggests that these surgical interventions were not limited to the extraction of the heart. The thorax and the abdomen of one of these subjects had been padded with vegetal matter. These examples were compared with that of Louis Bruslon du Plessis, who had been buried at the same time. The ribs of his perfectly preserved body had been sawn, his thorax, abdomen, and pelvis had been emptied and filled with layers of tow and vegetable matter for embalming, but his skull had not been opened. The meninges, that had fallen and begun decomposing, are visible in CT scans, as is the cerebral parenchyma that is resting against the posterior section of the cranium (Fig 10). At the cervical stage, the trachea and the larynx were visible. The trachea was cut at the cervico-thoracic junction. The spine was intact. At the chest level, the MSCT revealed that a thoracotomy had been realized, with a bilateral section of the sterno-costal cartilages. The thoraco-abdomino-pelvic cavities were filled with a vegetal material consisting in horizontal layers. The four subjects from the Jacobin convent (4/483, which represents 0.8% of the subjects found there) had thus all been embalmed.

## Discussion

2.7% (13/483) of the subjects from the convent in Rennes, in which a large number of nobles were buried in the 17^th^ century, showed signs of post-mortem surgical intervention. These included subjects from whom only the heart was extracted (4/483 or 0.8%), subjects whose hearts were removed and who were also embalmed (4/483 or 0.8%), and subjects who had undergone craniotomies (5/483 or 1%). We noted no significant distinction in either age (the practice involved no one younger than 5) or sex (7 males, 4 females). All of the embalmed subjects were found in valued areas of the convent, which was not the case for those who had been subjected to either heart extraction or craniotomies but had not been embalmed.

The considerable historical data that deals with funeral rites in Europe [3,33,34,4,35–37] focuses essentially on the nobility. The historians who draw on these data generally consider heart extraction and embalming to be coincidental [34,38], as was the case for the kings and queens of France [39,3,40]. They see the opening of the thoracic cage during the Renaissance as routinely practiced in combination with embalming, even though the primary sources on the matter are ambiguous [41]. The biological data on post-mortem interventions from the 13^th^ through the 18^th^ centuries come from a variety of sources: embalmed and well-preserved bodies randomly discovered [11,12,42], digs focused on the more or less well-preserved bodies of persons of prestige [43–45,13], isolated discoveries of bodies with no historical context [15,16,46], mounds of bones mixed together in ossuaries [8,9,47], and hearts contained in urns [48]. None of these studies established the incidence of these practices within the population, nor did any of them posit the hypothesis that heart extraction might have been performed other than in combination with some other procedure. Historians and archaeologists alike have consistently associated heart extraction with embalming. There do exist documented cases of isolated craniotomies, but the meaning ascribed to them oscillates between autopsy and embalming depending on the location of the discovery [49,10].

**Fig 11:**
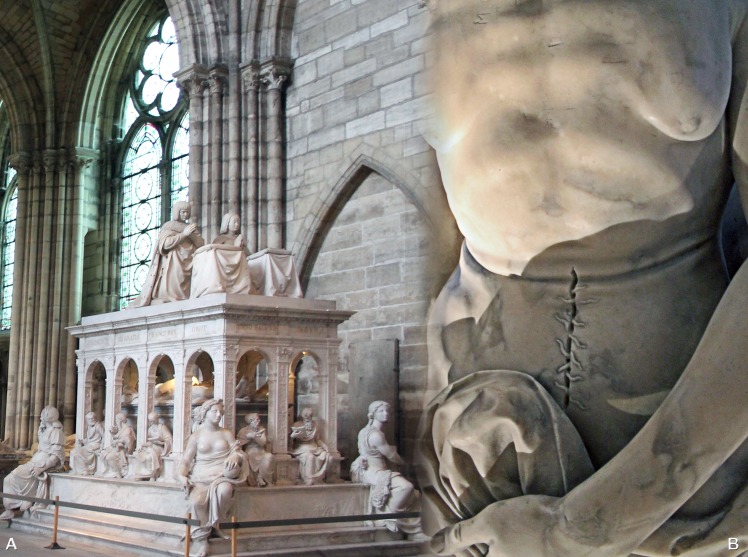
The funerary monument to Louis XII and Anne de Bretagne. (A) Basilica of Saint Denis. (B) Detail photograph of the incision on the Queen's abdominal wall.

In the world of art, the Basilica of Saint Denis just outside of Paris houses the 16^th^ century funerary monument to Anne de Bretagne and her husband, the King of France, Louis XII. They are shown on their deathbed, partly unclothed with a median, vertical incision in the abdominal-pelvic region that is stitched closed with coarse thread. Neither the location of the incision nor the surgical technique corresponds to what was observed on the two studied bodies, where the opening was made in the abdominal-thoracic region, with perpendicular incisions in the abdomen in the form of a cross (Fig 10). The technique of closing the incision with simple sutures is similar to the one used on Louise de Quengo. Whereas the manner in which Louise de Quengo's heart was extracted attests to considerable knowledge of thoracic anatomy, the technique for sewing the incision is not indicative of advanced surgical training (Fig 8). The operation might effectively have involved two people: a surgeon and his assistant. In the case of Louis Bruslon du Plessis, the opening was closed with a continuous suture, a more sophisticated technique occasionally described in medical treatises [50]. It was executed perfectly, which suggests the hand of a surgeon (Fig 11). The exceptional preservation of the teguments allows us for the first time to describe very precisely this surgical protocol.

Craniotomies and heart extractions not performed in combination with other procedures–the first such heart extraction we know of being that of Louise de Quengo–were carried out to remove an organ. They were not undertaken with the aim of preserving the body or even of presenting family members with a body that could be considered to be resting peacefully, which is the purpose of embalming today. We noted no desire whatsoever to preserve Louise de Quengo's body. The internal organs had not been cleaned, no padding had been introduced, the skull had not been cut into, and there were no incisions on the upper or lower limbs, as was often recommended in medical treatises of the period [51,50,52]. In the case of Louise and her husband, the idea was apparently to honor two religious sites of which they had been benefactors and, through their complementary burials–one spouse's heart buried with the other spouse's body–suggest the strength of their attachment to each other. Moreover, multiple resting places not only made it possible to honor several different religious orders, but also allowed for a greater number of prayers, which was especially important for believers who, since the Middle Ages, thought that prayer alone could help them avoid the purgatory for which they were destined and allow them to enter heaven. Removing just the heart also represented a middle ground between, on the one hand, the numerous funeral services for the body, the heart, the entrails, sometimes even the bones that had gained favor since the Crusades and, on the other, the integrity of the body advocated in the papal bull issued by Boniface VIII in 1299 [53], and of which Louise de Quengo was surely mindful given her religious attire and her association with a mendicant order. Embalming hearts was tied to the need to preserve them during the long period that preceded burial, which, in the case of Louise de Quengo's husband, was 7 years. During that time, they were hung in the church choir or in a side chapel that was later to house the coffin, except in those instances when they were put on display or stored in an armoire [40], as was the case for the hearts of the French kings in Val-de-Grace in Paris.

The reasons for craniotomies not carried out in conjunction with other procedures are less apparent. That said, the fact that half of them were found in the choir near lead urns containing hearts (3/5), lead coffins (4/5), and embalmed bodies (4/4) leads one to conclude that the procedure was more likely part of a ritual–the extraction of the brain–than a necessary step in an autopsy or an attempt to satisfy anatomical curiosity. We do not know where brains were placed, nor do we know whether brains and entrails were treated in some special way, as hearts were. We do know, however, that lead coffins, which had been known since the end of Roman times [54], were intentionally ostentatious, and also that they helped preserve even bodies that had not been treated, which was a sign of saintliness [55,56]. Coincident to these distinct extractions, there also existed cases of embalming that actually seem to be forerunners of the ways bodies are dealt with today, with an eye towards presenting them for viewing and delaying decomposition. The case of Louis Bruslon du Plessis, however, runs contrary to that impression, because an embalming product was applied to his face prior to his being wrapped in two cloth shrouds tightly held in place by a rope. Clearly, the point was to preserve the body. This point of view was quite different from the one that motivated those who ascribed to Louise de Quengo's thinking, and which clearly equated the Resurrection of the Flesh and the resurrection of flesh, a position that had been valorized by the Council of Trent, with which the Jesuits were very familiar.

## Conclusion

Despite the context of a Medieval religious unity crushed by the Reformation and Counter-Reformation, the goal of the Modern funerals of these characters was not only staged for judicial and / or political purposes (to hand down some power or inheritance) in which embalming would simply provide the temporary preservation of the body (material need). The study of the Jacobin convent in Rennes has demonstrated that Renaissance society's attitude towards burials favored maintaining age-old practices. As had been the case several centuries before, many nobles wished to be buried in prized areas of the church, but few of them chose to be buried in lead coffins, and only a minority of them opted for embalming. It is conceivable that this latter practice, which long before had been the privilege of royalty, had been revitalized by the Council of Trent's affirmation of the Resurrection of the Flesh. The absence of systematic presentation of the bodies also evokes the possibility of a trend linked to odours of sanctity, to the cult of saints and to rot proof corpses, suggested by the same Council. Other nobles, however, too few in number, preferred to have just their hearts removed, a more modest gesture that was more in line with the religious concept of the integrity of the body that had been promoted many centuries earlier. It allowed for multiple funeral services that could honor several different religious sites and, on a more intimate level, for couples to be reunited in death, a phenomenon that had until now not been noted. None of these practices suggests the approach to embalming that was to take hold in the 19th century. In this domain as in others, it would be another hundred years before the practice became secularized, with the 1776 edict outlawing burials in churches and then the French Revolution. One of the last hearts to be removed in France was that of Léon Gambetta (died in 1882). It was placed in the Pantheon in 1920, a veritable temple of republicanism dedicated to honoring great men [57]. It will take many more digs on the sites of the major cemeteries of the elite for us to establish the chronological evolution of these rites and the attitudes of Europeans towards them.
